# Development and Assessment of Multiple Illumination Color Fourier Ptychographic Microscopy for High Throughput Sample Digitization

**DOI:** 10.3390/s24144505

**Published:** 2024-07-12

**Authors:** Patrik Gilley, Ke Zhang, Neman Abdoli, Youkabed Sadri, Laura Adhikari, Kar-Ming Fung, Yuchen Qiu

**Affiliations:** 1 School of Electrical and Computer Engineering, University of Oklahoma, Norman, OK 73019, USA; patrik.w.gilley-1@ou.edu (P.G.); youkabed.sadri@ou.edu (Y.S.); 2 Stephenson School of Biomedical Engineering, University of Oklahoma, Norman, OK 73019, USA; 3 Department of Pathology, University of Oklahoma Health Sciences Center, Oklahoma City, OK 73104, USA; laura-adhikari@ouhsc.edu (L.A.); karming-fung@ouhsc.edu (K.-M.F.)

**Keywords:** multiplexed illumination, color FPM, scanning microscopy

## Abstract

In this study, we proposed a multiplexed color illumination strategy to improve the data acquisition efficiency of Fourier ptychography microscopy (FPM). Instead of sequentially lighting up one single channel LED, our method turns on multiple white light LEDs for each image acquisition via a color camera. Thus, each raw image contains multiplexed spectral information. An FPM prototype was developed, which was equipped with a 4×/0.13 NA objective lens to achieve a spatial resolution equivalent to that of a 20×/0.4 NA objective lens. Both two- and four-LED illumination patterns were designed and applied during the experiments. A USAF 1951 resolution target was first imaged under these illumination conditions, based on which MTF curves were generated to assess the corresponding imaging performance. Next, H&E tissue samples and analyzable metaphase chromosome cells were used to evaluate the clinical utility of our strategy. The results show that the single and multiplexed (two- or four-LED) illumination results achieved comparable imaging performance on all the three channels of the MTF curves. Meanwhile, the reconstructed tissue or cell images successfully retain the definition of cell nuclei and cytoplasm and can better preserve the cell edges as compared to the results from the conventional microscopes. This study initially validates the feasibility of multiplexed color illumination for the future development of high-throughput FPM scanning systems.

## 1. Introduction

In clinical practice, microscopic scanners have been widely used for various applications [1]. For example, frozen section tissue samples acquired during surgery are digitized quickly and transferred to the pathologist’s workstations for intraoperative diagnosis [2]. Pathologists in small hospitals located in rural areas need to send the digitized suspicious or ambiguous samples to specialty pathology laboratories for remote diagnosis [3,4]. In fine needle aspiration (FNA) biopsy, the collected samples are processed and digitized on-site immediately. Thus, the pathologists can remotely evaluate whether adequate samples are collected for the following diagnosis or not [5,6] (i.e., rapid on-site evaluation, ROSE [6,7]). As a result, scanning microscopy-based digital pathology demonstrates several advantages over conventional approaches [8].

One of the most important qualities of microscopic imaging systems today is their information throughput capacity. High information throughput is crucial in medical imaging fields such as digital pathology, as image acquisition speed is just as important as image spatial resolution [9]. However, conventional microscopic imaging systems are only capable of achieving either a high field-of-view (FOV) or high resolution on the captured images [10]. Clinical slide scanners compensate for resolution and FOV tradeoffs by using costly precision mechanical scanning systems to acquire a series of high-resolution, low FOV images of clinical specimens, which are stitched together to generate a whole slide image of acquired specimens [11]. Despite their effectiveness, current slide scanners are expensive and cumbersome, which significantly limits their wider applications in various clinical scenarios.

To overcome this limitation, Fourier ptychography microscopy (FPM) [10,12,13] is one promising solution. FPM is a computational imaging method that provides a low-cost approach to simultaneously achieve large FOV and high spatial resolution. FPM captures a series of low-resolution images of a specimen under a wide range of illumination angles generated through the use of an LED grid. A phase retrieval algorithm is utilized to computationally synthesize a high-resolution image that preserves the FOV [14] of the original objective lens [10,15,16]. Additionally, the computational nature of the FPM image reconstruction process provides for powerful tools such as defocus correction [10,17] and pupil function recovery [15]. However, FPM image reconstruction algorithms require a large number of raw low-resolution images to successfully reconstruct high-resolution images. Coupled with the need for longer exposure times for each captured image to counteract the weaker intensity of LED illumination, the FPM data acquisition process is very time intensive. This issue is exacerbated when colored images are needed, as the red, green, and blue channels need to be captured separately during conventional FPM data acquisition. This inhibits its utility in time sensitive applications such as in vivo imaging and high-volume imaging. Several strategies have been proposed [16,18,19,20,21,22,23] to mitigate this problem by reducing the number of raw images required for successful image reconstruction. One strategy is multiplexing the information recorded by the system, which can take two forms: angular multiplexing [16,18,19], which combines multiple LED positions per raw image, and spectral multiplexing [20,21], which combines multiple illumination wavelengths per raw image. However, to the best of the authors’ knowledge, few experiments have been conducted on combining the angular and spectral multiplexing together to further enhance the efficiency of sample digitization.

For this purpose, we built a multiplexed FPM-based microscope equipped with a color camera, which aims to achieve angle-spectrum multiplexing data acquisition. A standard USAF 1951 resolution target was reconstructed to assess the system imaging performance, and the clinical potential was then validated by imaging and reconstructing high-resolution images of several clinical samples. More details are presented in the following sections.

## 2. Materials and Methods

### 2.1. Multiplexed Fourier Ptychography

Fourier ptychography microscopy (FPM) is a computational imaging process that can enhance the performance of low-power microscopes using phase retrieval and synthetic aperture techniques [10]. The primary difference between conventional and multiplexed FPM methods is in their data acquisition strategies. Instead of relying on the sequential single monochrome LED activation process used in conventional FPM, multiplexed FPM activates multiple triple color LEDs per image acquisition. Accordingly, when capturing a raw low-resolution image, the sample, modeled using the complex transmittance function O(r), is illuminated by a unique plane wave. This plane wave is emitted by multiple triple color (i.e., red, green, blue) LEDs, and each single color is assumed to be spatially coherent. Thus, this plane wave can be considered as a combination of several single-color monochrome exit waves. Each single wave is from one LED generating a certain illuminating wavelength h (i.e., RGB). Meanwhile, traditional objective lenses act as circular low-pass filters on the sample’s frequency spectrum R(k), with a cutoff frequency of 2πNA/λ, where λ is the illumination wavelength and NA is the numerical aperture of the objective lens. For each single LED m, the center of R(k) in the Fourier domain will shift by $k_{m}$ when the sample is illuminated by a plane wave with an incidence angle of ($\theta_{x},\theta_{y})$, where $k_{m}=(sin\;\left(\theta_{xm}\right)\;/\lambda ,sin\;\left(\theta_{ym}\right)\;/\lambda )$. Therefore, a multiplexed FPM optical system can record a much wider range of frequency information from the sample spectrum because of the range of incidence angles that an LED grid is capable of.

Accordingly, at the image plane, the raw image intensities recorded are the result of the sample exit wave that is filtered by the objective lens pupil function P(k). As discussed above, this exit wave contains the information of multiple color wavelengths. For each individual color, the wave intensity can be written as follows:

(1) $$I_{n}\left(r\right)=\sum_{m\in L_{n}}i_{m}\left(r\right)=\sum_{m\in L_{n}}{\left|F^{-1}\left[O\left(k-k_{m}\right)*P\left(k\right)\right]\right|}^{2}$$

where $F^{-1}$ is the inverse 2D Fourier transform, k is the spatial wavenumber, $L_{n}$ is the set of LED indices associated with the nth image, $i_{m}$ represents the intensity at the image plane from a single LED, $k_{m}$ is the incident plane wave vector, and m is the activated LED index. The microscope system’s NA is synthetically extended by the maximum LED illumination angle, according to [24]: ${NA}_{sys}={NA}_{obj}+sin\;\left(\theta_{max}\right)$, where $\theta_{max}$ is the maximum incident angle of the LED grid (assuming the illumination medium is air).

### 2.2. Experimental Setup

**Figure 1:**
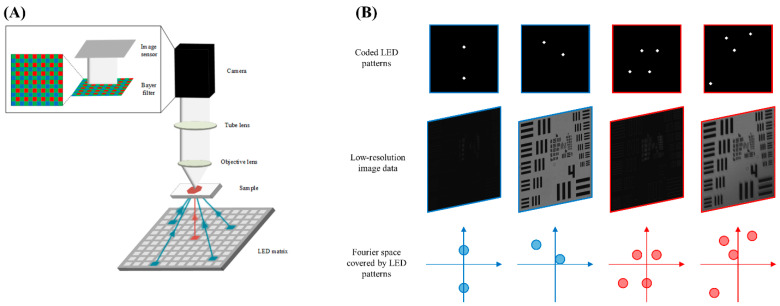
Fundamentals of FPM microscope technology. (**A**) Schematic of experimental FPM microscope demonstrates the microscope using conventional one-LED illumination (red arrow) and multiplexed four-LED illumination (blue arrows). (**B**) Samples of multiplexed illumination patterns. Samples represent two-LED (blue) and four-LED (red) illumination patterns. The top row of images represents positions of activated LEDs on the grid for each image. The middle row shows the raw low-resolution images captured using the activated LEDs. The bottom row illustrates the Fourier space subregions sampled by the captured raw images.

In order to validate the performance of the multiplexed FPM method with color imaging, we constructed our FPM imaging system using components from Thorlabs’ Cerna modular microscopy platform (Figure 1). The color camera used in the imaging system was a FL20 color cooled CMOS camera (Tuscen, Fuzhou, China). An overall system magnification of 3× was achieved by equipping a 4×/0.13 NA Plan Fluor objective lens (Nikon, Tokyo, Japan) and a 150 mm achromatic doublet (AC508-150-A, Thorlabs, Newton, NJ, USA) tube lens. A 32 × 32 programmable color LED array (Adafruit, New York City, NY, USA), was installed ~72 mm below the sample stage to provide the angled illumination required by the FPM imaging method. Each LED position on the array consisted of three different internal LEDs that were designed for red light (0.63 µm), green light (0.53 µm) and blue light (0.47 µm) illumination, respectively. White light illumination was generated from each LED position by simultaneously activating all three of its internal LEDs. Our experiment utilized a centralized 15 × 15 LED matrix, which resulted in an illumination NA of ~0.362. Therefore, the estimated synthetic NA of the FPM imaging system was ~0.492. The FL20 color camera used in this study achieved color imaging via the use of a Bayer filter. This filter sub-sampled each color channel when capturing an image, resulting in 75%, 50%, and 75% of the pixels in the R, G, and B color channels being empty, respectively. Although the number of empty pixels is different across the three channels, it will not significantly affect the quality of the reconstructed images, as sampled raw images contain adequate information from the objective lens. The FPM image reconstruction process for each image was restricted to the non-empty pixels of each raw low-resolution image when reconstructing each color channel high-resolution image. Additionally, the conventional FPM reconstruction algorithm employed an intensity correction method that applied a scaling factor to each real-space representation update after the initial iteration of the reconstruction algorithm. This scaling factor adjusted the intensity of each low-resolution image to account for the uncertainty of each LED’s illumination intensity [25]. FPM defocus correction factors were determined for each FPM illumination pattern, with individual correction factors for each color channel within those illumination patterns.

### 2.3. Multiplexed Illumination Pattern Design and FPM Image Reconstruction

A crucial step in using multiplexed FPM is generating the LED patterns used to image the sample. It is important to ensure that LED positions are not oversampled while preserving the image overlap required by FPM image reconstruction algorithms to successfully converge on a solution [26]. This ensures that each LED position is sampled once even after reducing the total amount of acquired raw images. Therefore, the LED pattern generation process is governed by several important rules. The LED positions for each image are selected randomly using uniform probability. Once an LED position is assigned to an image, it is excluded from being selected for all other images in the image set. These rules will generate LED positions for an image set of $\frac{N_{LED}}{M}$, where $N_{LED}$ is the total number of LEDs in the LED grid, and M is the total number of LEDs per image [16]. In order to ensure that there were enough image positions to match the number of images produced by conventional FPM, the LED pattern generation process was repeated M times.

Once a full set of raw low-resolution images is collected, they can be used to reconstruct a high-resolution image. As the image reconstruction process is done in the complex Fourier domain, it becomes necessary to have both intensity and phase information for each raw image. Given that the captured raw image patterns only record intensity information, the FPM reconstruction process employs a phase retrieval algorithm to recover the missing phase information from the raw low-resolution images. The phase retrieval algorithm utilized in this study was a modified version of the embedded pupil function recovery (EPRY) algorithm [15,16]. The EPRY algorithm computationally fuses the information in each image to recover phase information from the sample while simultaneously optimizing the pupil function to remove the effects of lens aberrations from the final high-resolution reconstruction. The first step of the reconstruction process initializes a guess of the high-resolution image using the square root of the brightest raw image. The sample spectrum is divided into subregions that correspond to each acquisition n. For the nth obtainment, the corresponding sample spectrum is converted to its real space representation by $\psi_{m}^{\left(i\right)}\left(r\right)=F^{-1}\left[O^{\left(i\right)}\left(k-k_{m}\right)*P^{\left(i\right)}\left(k\right)\right],\;m\in L_{n}$ where m represents the mth LED activated in the nth data acquisition. The amplitude of $\psi_{n}^{\left(i\right)}(r)$ is then replaced by the recorded raw image intensity $I_{raw}^{n}$ according to the EPRY intensity constraint $\phi_{m}^{\left(i\right)}\left(r\right)=\sqrt{\frac{I_{n}\left(r\right)}{\sum_{m\in L_{n}}{\left|\psi_{m}^{\left(i\right)}\left(r\right)\right|}^{2}}}\psi_{m}^{\left(i\right)}\left(r\right),\;m\in L_{n}$. The revised real space representation is then used to update the appropriate sample spectrum and pupil function subregions based on the Fourier transform of the real space representation $\Phi_{m}^{\left(i\right)}(k)=F^{-1}\left[\phi_{m}^{\left(i\right)}(r)\right]$ $\Phi_{n}^{\left(i\right)}(k)=F^{-1}\left[\phi_{n}^{\left(i\right)}(r)\right]$. The iterative reconstruction process uses a Gauss–Newton approach to converge to the final high-resolution image [16]. This process is repeated for several iterations to ensure that the image reconstruction process converges on an optimal estimate of the final high-resolution image.

### 2.4. Multiplexed FPM System Performance Evaluation

We initially evaluated the multiplexed FPM color imaging strategy by imaging a standard USAF 1951 resolution target (Newport, Irvine, CA, USA) under white light illumination. The USAF 1951 target was imaged using conventional, two-LED multiplexed, and four-LED multiplexed FPM illumination, which are referred to as one-LED, two-LED, and four-LED illumination for the rest of this paper, respectively. The reconstructed R, G, and B color channel high-resolution images of both multiplexed FPM configurations were compared with the reconstructed color channel images of conventional FPM in order to subjectively evaluate their imaging quality and resolution. Additionally, the reconstructed USAF 1951 target images were used to generate modulation transfer function (MTF) curves [27] for each color channel of the one-LED, two-LED, and four-LED illumination methods. The MTF curve contrast values were measured from select bar patterns on the reconstructed USAF 1951 target images, with contrast values calculated using the equation: $C=\frac{I_{max-I_{min}}}{I_{max}+I_{min}}$, where $I_{max}$ and $I_{min}$ are the maximum and minimum pixel values of each selected bar pattern, respectively [28]. These contrast values were used to generate smoothed MTF curves from 0 lp/mm to the cutoff frequency of each FPM illumination method through the application of a curve fitting algorithm [29].

We imaged several clinical samples to assess the utility of our system in clinical applications. An H&E-stained ovarian cancer tissue sample was first imaged to assess the performance of our system in color imaging. The reconstructed images from the different FPM illumination methods were compared with a reference image captured using a conventional microscope equipped with a 20×/0.4 NA objective lens. The white balance of the reconstructed H&E-stained sample images was set by normalizing the color channels to achieve a uniform mean value [30]. Additionally, a set of metaphase chromosomes [28,31] were imaged under green light illumination ($\lambda_{green}$ = 530 nm), which was acquired from leukemia blood samples. The performance of each FPM illumination method used in this study was assessed by comparing the quality of the band patterns resolved in the reconstructed images. Both samples were prepared in our medical center using standard clinical protocols.

## 3. Results

**Figure 2:**
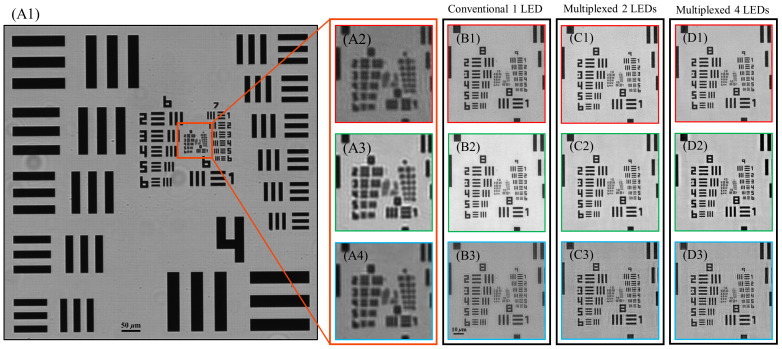
Image data of the USAF 1951 resolution target. The borders of all individual color channel images are highlighted with the color channel they represent. (**A1**) Raw image of the 1951 USAF resolution target captured by the 4×/0.13 NA objective lens under white light illumination using only the central LED. (**A2**–**A4**) Expanded images of the red, green, and blue channels of the outlined ROI in (**A1**). All missing pixels were filled in via interpolation. (**B1**–**B3**) Red, green, and blue channels of the image reconstructed using conventional one-LED illumination, respectively. (**C1**–**C3**) and (**D1**–**D3**) are the reconstructed results of two-LED and four-LED multiplexed illumination, respectively.

Figure 2 shows the reconstructed image of our USAF 1951 resolution target. Figure 1(A1) illustrates the raw low-resolution image data captured by our system when utilizing only the central LED, and Figure 2(A2–A4) depicts the red, green, and blue channels of the central region of Figure 1(A1), respectively. These results indicate that the 4×/0.13 NA lens can resolve frequencies up to approximately 228.1 lp/mm (Group 7 Element 6). Figure 2(B1–B3) depicts the red, green, and blue channels of the high-resolution image reconstructed using one-LED illumination. All three color channels are consistently able to resolve frequencies up to 724.1 lp/mm (Group 9 Element 4). The green and blue color channels in Figure 2(B2,B3) are capable of resolving frequencies up to 912.3 lp/mm (Group 9 Element 6). The blue channel shows markedly more artifacts than the other color channels in the reconstructed image, despite retaining a high resolving power. Images reconstructed using two-LED and four-LED illumination are shown in Figure 2(C1–C3) and Figure 2(D1–D3), respectively. Both multiplexed illumination methods also produced a greater degree of noise around the bar patterns in these color channels, which was much more pronounced in the four-LED reconstructed image. The additional noise in the blue channels for the two-LED and four-LED reconstructed images reduced their maximum resolvable frequency from 912.3 lp/mm (Group 9 Element 6) to 812.7 lp/mm (Group 9 Element 5).

**Figure 3:**
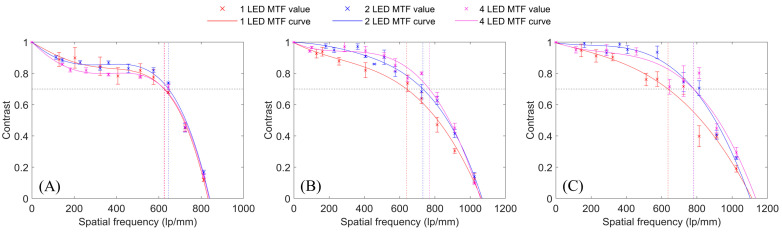
MTF curves measured from FPM reconstructions from conventional illumination, two-LED multiplexed illumination, and four-LED multiplexed illumination. (**A**) Red channel. (**B**) Green channel. (**C**) Blue channel.

Figure 3A–C shows the MTF curves for the one-LED, two-LED, and four-LED illumination methods for the R, G, and B channels, respectively. The MTF curves indicate that the tested FPM illumination methods produced very similar cutoff frequencies in all three color channels. Both two-LED and four-LED multiplexed FPM configurations produced consistently better contrast than the conventional FPM configuration in the green and blue channels. In the red channel, two-LED illumination curve produced better contrast values than conventional one-LED illumination curve for spatial frequencies of approximately 240 lp/mm and above, while four-LED illumination noticeably underperformed one-LED illumination in the red channel for frequencies of approximately 602 lp/mm and less. When comparing the performance of the two-LED and four-LED configurations, it can be observed that two-LED illumination outperformed four-LED illumination in the red channel. However, the two-LED and four-LED illuminations were comparable in the green and blue channels. Two-LED illumination produced higher contrast values up to 782 lp/mm in the blue channel, but four-LED illumination produced better contrast values between 328 lp/mm and 943 lp/mm in the green channel.

**Figure 4:**
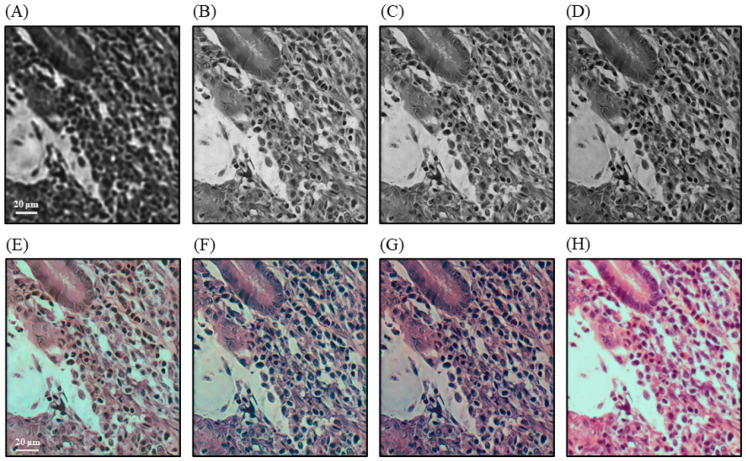
Images of an ovarian cancer pathology slide sample. (**A**) Raw image captured by a 4×/0.13 NA objectives lens using the central LED. (**B**–**D**) Green channels of the images reconstructed using one-LED, two-LED and four-LED illumination, respectively. (**E**–**G**) Color image reconstructed using one-LED, two-LED, and four-LED illumination, respectively. (**H**) Color image captured under a microscope using a 20×/0.4 NA objective lens.

Figure 4 shows images of the H&E ovarian cancer pathology slide reconstructed using one-LED, two-LED, and four-LED illumination. Figure 4A depicts a raw low-resolution image captured using the 4×/0.13 NA objective lens, which is too blurry to resolve any details within the cells themselves. Figure 4B–D shows the green channel of the high-resolution image reconstructed using one-LED, two-LED, and four-LED illumination, respectively. The four-LED reconstructed image was slightly noisier than the one-LED and two-LED reconstructed images, but all three illumination methods achieved similar results in terms of resolving cell nuclei and cytoplasm. The full color reconstructions of the one-LED, two-LED, and four-LED illuminations are shown in Figure 4E–G, respectively. The multiplexed illumination method reconstructed images were of comparable quality with the one-LED reconstructed image (Figure 4B), preserving the definition of cell nuclei and cytoplasm without adding any significant noise or artifacts. Figure 4H contains an image of the ovarian cancer pathology slide sample area captured under a conventional microscope equipped with a 20×/0.4 NA objective lens. Both multiplexed FPM illuminations were better able to retain the edges of these difficult nuclei than conventional FPM illumination did.

**Figure 5:**
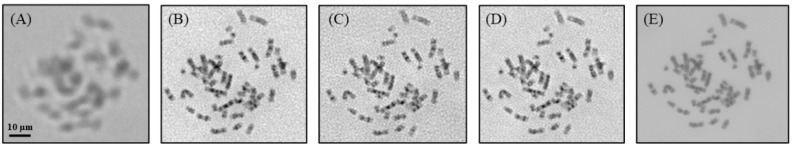
Images of a leukemia blood sample set of chromosomes. (**A**) Raw image captured by a 4×/0.13 NA objective lens using the central LED. (**B**–**D**) Reconstructed amplitude images of the chromosomes using one-LED, two-LED, and four-LED illuminations, respectively. (**E**) Chromosome image captured using a 20×/0.4 NA objective lens.

Figure 5 shows the FPM imaging results of metaphase chromosomes from a leukemia blood sample, which were imaged under green light illumination. An important target for karyotyping chromosomes is the band patterns of each chromosome, as band patterns are unique to each chromosome [32]. Figure 5A contains a raw low-resolution image of the metaphase cell chromosomes, captured by the 4×/0.13 NA objective lens using the central LED. Only the outlines of the chromosomes can be observed in this image; no details about the chromosome bands can be distinguished. Figure 5B–D depicts the amplitude images of the blood chromosomes using one-LED, two-LED, and four-LED illumination, respectively. All of these results maintained clear edges and sharp band patterns on the chromosomes. Figure 5E shows an image of the sampled chromosomes captured using a conventional microscope equipped with a 20×/0.4 NA objective lens under green light illumination. All three reconstructed images achieved comparable sharpness in resolving the chromosome band patterns, producing better sharpness than the conventional 20×/0.4 NA objective lens imaging results.

## 4. Discussion

In this paper, we developed and initially evaluated a multiplexed FPM color imaging system, using both the standard resolution target and clinical samples. As compared to single illumination FPM, the major advantage of the multiplexed color FPM technique is that it can vastly enhance image acquisition efficiency. Since multiple LEDs will be turned on for each single image acquisition, the sample illumination intensity will increase multiple times, resulting in a significant reduction in exposure time requirements. For example, when four-LED illumination is applied, the exposure time can be reduced by up to ¼. Furthermore, the frame rate can correspondingly increase by up to four times. In addition to the improvements on image acquisition speed, the number of raw images required for successful reconstruction can potentially be reduced by up to ¼, given that one acquired raw image contains four different spectrum regions in our example. (e.g., from 225 to 57 in our configuration). Additionally, one color camera was employed in this study, which can simultaneously acquire red, green, and blue channel signals together, further reducing the total number of raw images by a factor of three. Taking all of these factors into account, four-LED color FPM has the potential to reduce the total acquisition time up to 48 times as compared to the conventional single-LED grayscale FPM. This efficiency improvement can be further enhanced by illuminating more than four LEDs at the same time.

Second, the multiple illumination FPM results achieved a comparable image quality as compared to the single illumination results. The two-and four-LED reconstructions contain more artifacts, which can be attributed to the superposed spectra on the raw images. During the reconstruction process, only one sample spectrum subregion is restored at a time when processing a raw image. The other spectrum subregions will be considered as noise. For example, when reconstructing four-LED FPM image data, three of the four subregions in each raw image will be treated as noise to the subregion currently being processed by the reconstruction algorithm. Due to these issues, multiple illumination FPM reconstructions will typically have a lower signal to noise ratio than conventional FPM reconstructions. However, these artifacts did not significantly impact the reconstructed clinical samples. This is partially due to the fact that these artifacts are concentrated at the high frequency band, but also because most of the useful biological structures are distributed in the range of the middle frequencies.

This proposed technology can be used to develop a low-cost microscopic scanner with high scanning speed and adequate spatial resolution, which has widespread potential applications in current pathology labs. For example, in the intraoperative diagnosis of lung neoplasms [33], the frozen sections of the acquired samples are processed and stained on-site. However, pathology labs may not be conveniently located near the surgical suite, as pathology services are more and more consolidated in modern hospital systems. In current practice, one pathologist has to be physically present on-site for initial diagnosis of the tissue samples. More importantly, he/she needs to operate the digital microscope and demonstrate the suspicious regions of interest to the off-site thoracic pathologist for remote diagnosis. Current commercial whole slide scanners can avoid the need for an on-site pathologist, but they have large footprint and high system costs, which may not be affordable for most hospitals. Our FPM-based scanner, however, can be developed based on low-cost components and computers [34] to achieve similar performance to current whole slide scanners at a cost similar to standard digital microscopes while maintaining the portability of the digital microscopes. Thus, only technicians are needed at these locations to prepare the sample and operate the scanner. All the pathologists can work at distant suites for remote diagnosis, and their working efficiency can be significantly enhanced. Meanwhile, this technology may also present significant improvements in other commonly used approaches such as ROSE, reducing the need for experienced on-site pathology personnel [35].

Despite the encouraging results, we acknowledge that this study has the following limitations. First, only two- and four-LED illuminations were used in this study; other possible coding patterns were not investigated. The raw images were only acquired under 4×/0.13 NA lens to reconstruct 20×/0.40; high NA multiple LED FPM was not explored [24,36]. Second, we only evaluated the reconstructed images on H&E tumor tissues and analyzable chromosome cells. More histology and cytology samples should be evaluated to further validate the robustness of our system. Third, the reconstruction is based on conventional model-based algorithms; AI-based models [37] were not explored in this study. The learning-based code pattern optimization should also be investigated in the future [38,39]. Fourth, all the reconstructed results were based on a full set of 225 raw images. We did not evaluate the impact of reducing raw images on the multiplexed FPM illumination reconstructions, although it has the potential to further enhance the image acquisition efficiency. Fifth, despite the high frequency artifacts, the MTF still showed similar or even better cut-off frequencies for the two- and four-LED results. This implies that MTF curves may not properly reflect the generated artifacts in this application. It would be worthwhile to investigate more effective tools for evaluating the quality of multiplexed FPM reconstructions.

## 5. Conclusions

Multiple LED illumination FPM has the potential to significantly reduce the image acquisition time, thus further enhancing slide scanning efficiency with low modality costs. This investigation provides meaningful information for the development of a portable, low-cost whole slide digitizer for future clinical applications.

## Data Availability

Data are contained within the article.
